# ICTV Virus Taxonomy Profile: *Qinviridae* 2023

**DOI:** 10.1099/jgv.0.001905

**Published:** 2023-10-12

**Authors:** Yuri I. Wolf, Eugene V. Koonin, Mart Krupovic, Jens H. Kuhn

**Affiliations:** ^1^​ National Center for Biotechnology Information, National Library of Medicine, National Institutes of Health, Bethesda, MD 20894, USA; ^2^​ Institut Pasteur, Université Paris Cité, Archaeal Virology Unit, Paris 75015, France; ^3^​ Integrated Research Facility at Fort Detrick, National Institute of Allergy and Infectious Diseases, National Institutes of Health, Fort Detrick, Frederick, MD 21702, USA

**Keywords:** ICTV Report, *Qinviridae*, Taxonomy, yingvirus

## Abstract

*Qinviridae* is a family of negative-sense RNA viruses with genomes of 7.3–8.2 kb that have been associated with crustaceans, insects, gastropods, and nematodes. The qinvirid genome consists of two segments, each with at least one open reading frame (ORF). The large (L) segment ORF encodes a large protein containing an RNA-directed RNA polymerase (RdRP) domain. The small (S) segment ORF encodes a nucleocapsid protein. This is a summary of the International Committee on Taxonomy of Viruses (ICTV) Report on the family *Qinviridae*, which is available at ictv.global/report/qinviridae.

## Virion

Unknown.

## Genome

**Fig. 1.**
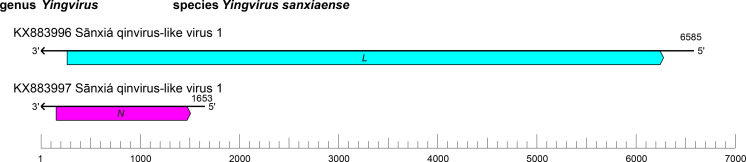
Genome organisation of a representative qinvirid, Sānxiá qinvirus-like virus 1. ORFs are coloured according to the predicted protein function (*L*, large protein gene encoding an RNA-directed RNA polymerase domain; *N*, gene encoding a nucleocapsid protein).

The qinvirid genome comprises two segments of linear negative-sense RNA with a total length of 7.3–8.2 kb (large (L) segment : 5.6–6.6 kb; small (S) segment : 1.6–1.9 kb) [1–5] (Table 1, Fig. 1). The L segment ORF encodes an RdRP with a GDP:polyribonucleotidyltransferase (PRNTase) domain that is related to that of viruses in the order *Mononegavirales*, suggesting a similar capping mechanism. The S segment ORF (*N*) encodes a nucleocapsid protein structurally related to the homologs encoded by mononegavirals, in particular paramyxovirids.

**Table 1.** Characteristics of members of the family *Qinviridae*

Example	Sānxiá qinvirus-like virus 1 (L:KX883996; S: KX883997), species *Yingvirus sanxiaense*, genus *Yingvirus*
Virion	Unknown
Genome	7.3–8.2 kb of bisegmented negative-sense RNA
Replication	Unknown
Translation	Unknown
Host range	Decapod crustaceans; blattodean and dipteran insects; gastropods; nematodes
Taxonomy	Realm *Riboviria*, kingdom *Orthornavirae*, phylum *Negarnaviricota*, class *Chunqiuviricetes*, order *Muvirales*; the family includes the genus *Yingvirus* and >7 species.

## Replication

Unknown

## Taxonomy

**Fig. 2.**
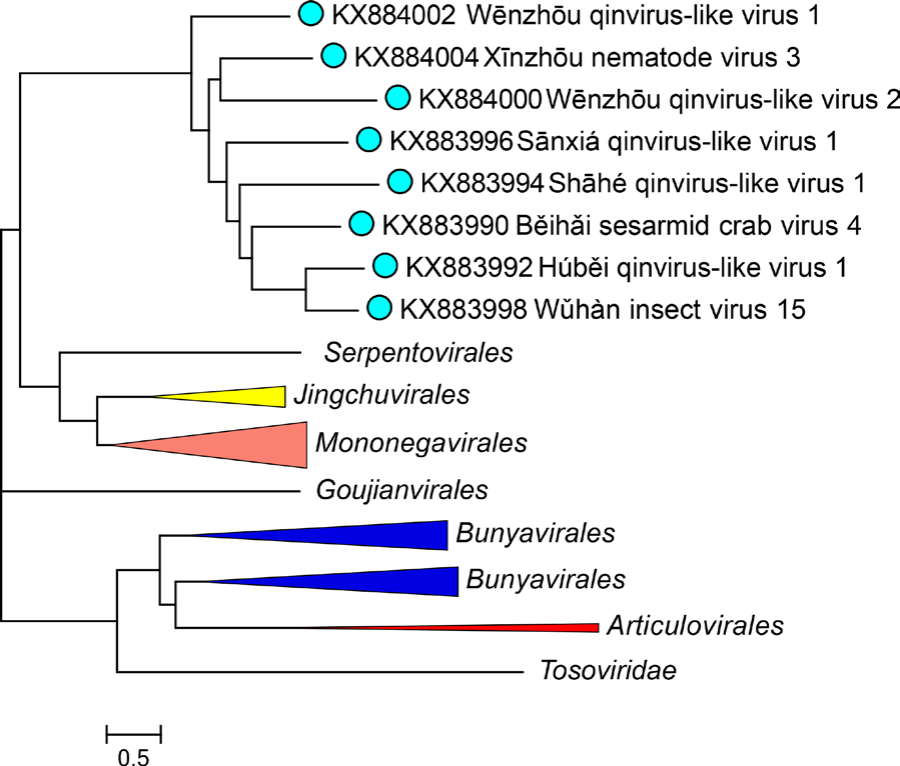
Phylogenetic relationships of qinvirids. A phylogenetic tree was reconstructed for an alignment of the RdRP core domains of the ICTV Virus Metadata Resource (VMR) set of *Negarnaviricota* using the FastTree programme. Branches for other taxa are collapsed to order taxon; the complete tree is available in the full *Qinviridae* ICTV Report.

Current taxonomy: ictv.global/taxonomy. The family *Qinviridae* includes the genus *Yingvirus* and multiple species for viruses that infect crustaceans, insects, gastropods, and nematodes. Qinvirids are most closely related to other viruses in the subphylum *Haploviricotina*, such as aspivirids, jingchuvirals, mononegavirals, and yuevirids (Fig. 2).

## Resources

Full ICTV Report on the family *Qinviridae*: ictv.global/report/qinviridae.
